# Postoperative diagnostic potentials of median nerve strain and applied pressure measurement after carpal tunnel release

**DOI:** 10.1186/s12891-019-3033-y

**Published:** 2020-01-11

**Authors:** Yuichi Yoshii, Wen-lin Tung, Hiroshi Yuine, Tomoo Ishii

**Affiliations:** 1 Department of Orthopaedic Surgery, Tokyo Medical University Ibaraki Medical Center, 3-20-1 Chuo, Ami, Tokyo, 300-0395 Japan; 2 Department of Occupational Therapy, Ibaraki Prefectural University of Health Sciences, Ami, Japan; 3 Department of Rehabilitation, Tokyo Medical University Ibaraki Medical Center, Ami, 300-0395 Japan

**Keywords:** Median nerve, Strain, Pressure, Carpal tunnel syndrome, Elastography, Ultrasound

## Abstract

**Bakground:**

The objective of this study is to investigate the prognostic values of median nerve strain and applied pressure measurement for the assessment of clinical recovery after carpal tunnel release.

**Methods:**

Forty-five wrists, from 45 idiopathic carpal tunnel syndrome patients who treated with open carpal tunnel release, were evaluated by ultrasound. Median nerve strain, pressure applied to the skin, and ratio of pressure-strain were measured at the proximal part of the carpal tunnel. In addition, distal latencies in the motor and sensory nerve conductions studies and cross-sectional area of median nerve were measured. The parameters were compared before and after the open carpal tunnel release. According to patient recovery, the receiver operating characteristic curves were generated to evaluate the prognostic values of the parameters. The areas under the receiver operating characteristic curves were compared among parameters.

**Results:**

There was a significant increase in the median nerve strain, and significant decreases in the pressure applied to the skin and ratio of pressure-strain after carpal tunnel release (*P* < 0.01). There were significant decreases in the distal latencies and the cross-sectional area after carpal tunnel release (*P* < 0.01). The areas under the curves were 0.689, 0.773, 0.811, 0.668, 0.637, and 0.562 for the pressure, strain, pressure-strain ratio, motor latency, sensory latency, and area, respectively.

**Conclusions:**

The results suggest that elasticity of the median nerve and pressure around the nerve recover quickly after carpal tunnel release. Pressure-strain ratio was the most reliable parameter to reflect clinical recovery. The measurement of strain and applied pressure can be useful indicators to evaluate effectiveness of the carpal tunnel release.

**Trial registration:**

Registered as NCT04027998 at ClinicalTrials.gov. Retrospectively registered on July 22, 2019.

## Background

Ultrasound elastography is a clinical examination that visualizes tissue stiffness [1–5]. As soft tissue pathological conditions result in changes in stiffness, elastography enables the differentiation of affected from normal tissue for diagnostic applications. Recently, the clinical applications of ultrasound elastography have expanded [6–9]. In musculoskeletal disorders, it was reported that elastography can detect pathological changes in tendons, nerves and muscle structures [10–12]. In the diagnosis of carpal tunnel syndrome (CTS), some studies reported that measurements of median nerve strain were useful. Lower strains of the median nerve were found in CTS patients compared with normal subjects [12, 13]. In order to determine the stiffness of deep tissue by elastography, it is necessary to apply an appropriate pressure from the body surface through a transducer. As previous studies did not consider applied pressure to the target structures, it was difficult to use the parameters quantitatively.

In a previous study, we developed a method to evaluate the relations of median nerve strain and applied pressure to give a quantitative assessment in the diagnosis of CTS [14]. It was found that there were lower strain and higher applied pressure values in CTS patients compared with normal subjects. Because pressure inside the carpal tunnel is higher in CTS patients [15–21], the force of repulsion during strain measurement may be higher. On the other hand, some reports showed a decrease in the carpal tunnel pressure after carpal tunnel release [15–17, 22, 23]. Generally, the measurement of carpal tunnel pressure requires invasive techniques. Thus, the development of a non-invasive method to show the changes in median nerve strain and internal pressure after carpal tunnel release may be useful to evaluate treatment effectiveness. In this study, we hypothesized that the strain and applied pressure may differ before and after carpal tunnel release in the CTS patients. The aim of this study was to evaluate the strain and applied pressure of the median nerve before and after carpal tunnel release in CTS patients. In addition, associations between clinical recovery and ultrasound parameters were evaluated, and postoperative diagnostic values were compared with electrophysiological and morphological parameters.

## Methods

This was a case-control study (level of evidence: level III). This study has been approved by the institutional review board (No. 14–5). This study was registered as NCT04027998 at ClinicalTrials.gov. Forty-five wrists of 45 idiopathic CTS patients who treated with open carpal tunnel release (32 females and 13 males; age range 37–89, mean age 66.8 years) were evaluated by ultrasound. Written informed consent was obtained from all study participants. All patients confirmed negative for secondary causes of CTS including diabetes, gout, rheumatoid arthritis, osteoarthritis, renal failure, previous traumatic injuries to the arm. CTS was diagnosed by characteristic clinical symptoms and delays in nerve conduction studies. Clinical symptoms are: numbness and pain in the hands exacerbated in the early morning or night, exacerbation when using the hands, objective sensory disorder corresponding to the median nerve area (especially ring-finger splitting), positive for Phalen’s test and/or Tinel’s sign, muscle weakness of the thumb abductor and thenar muscle atrophy. The first ultrasound evaluations were performed during the initial diagnostic process, one to three months before surgery. The nerve conduction studies were performed at the same time with ultrasound evaluations. After evaluating ultrasound image with an intact carpal tunnel, open carpal tunnel release was performed. After three months of the surgery, ultrasound and nerve conduction studies were performed again. The clinical recovery of the patient’s symptoms were evaluated by the changes of clinical symptoms before and after three months of the surgery. Based on each patient’s changes of the symptoms, we divided them into four groups: excellent, no symptom or remarkable improvement of pain and numbness with no restrictions on daily activity; good, slight improvement of pain and numbness with some restrictions on daily activity; fair, no changes in pain and numbness; and poor, worsening of pain or numbness.

### Median nerve strain and applied pressure measurement

Median nerve strain and applied pressure were measured with a pressure monitor ultrasound system. The details of the apparatus were described previously [14]. Vertical movement of transducer is adjustable from 0.1 mm to 3.0 mm by a programmed controller. A strain gauge sensor capable of measuring dynamic compression force was set between the transducer holder and the motor. The examination table was made so that the transducer could be placed at the wrist crease level of the subject. Using the placement adjuster, the height of the transducer to wrist was adjusted.

**Fig. 1:**
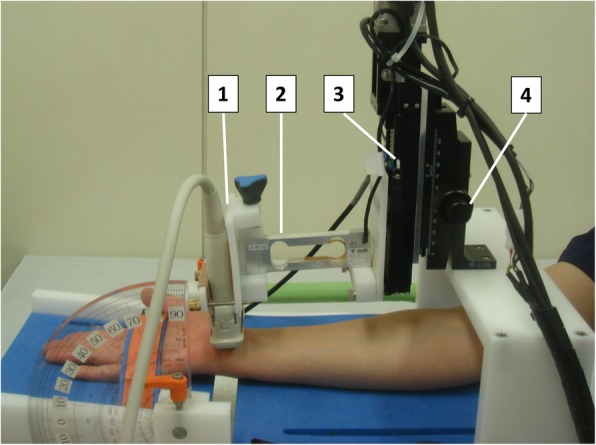
Setup for strain and pressure measurement. The participants were asked to sit and place their forearm on a table with the palmar side up. 1. Transducer holder, 2. Pressure sensor, 3. Motor for the compression-release cycle, 4. Placement adjuster

**Fig. 2:**
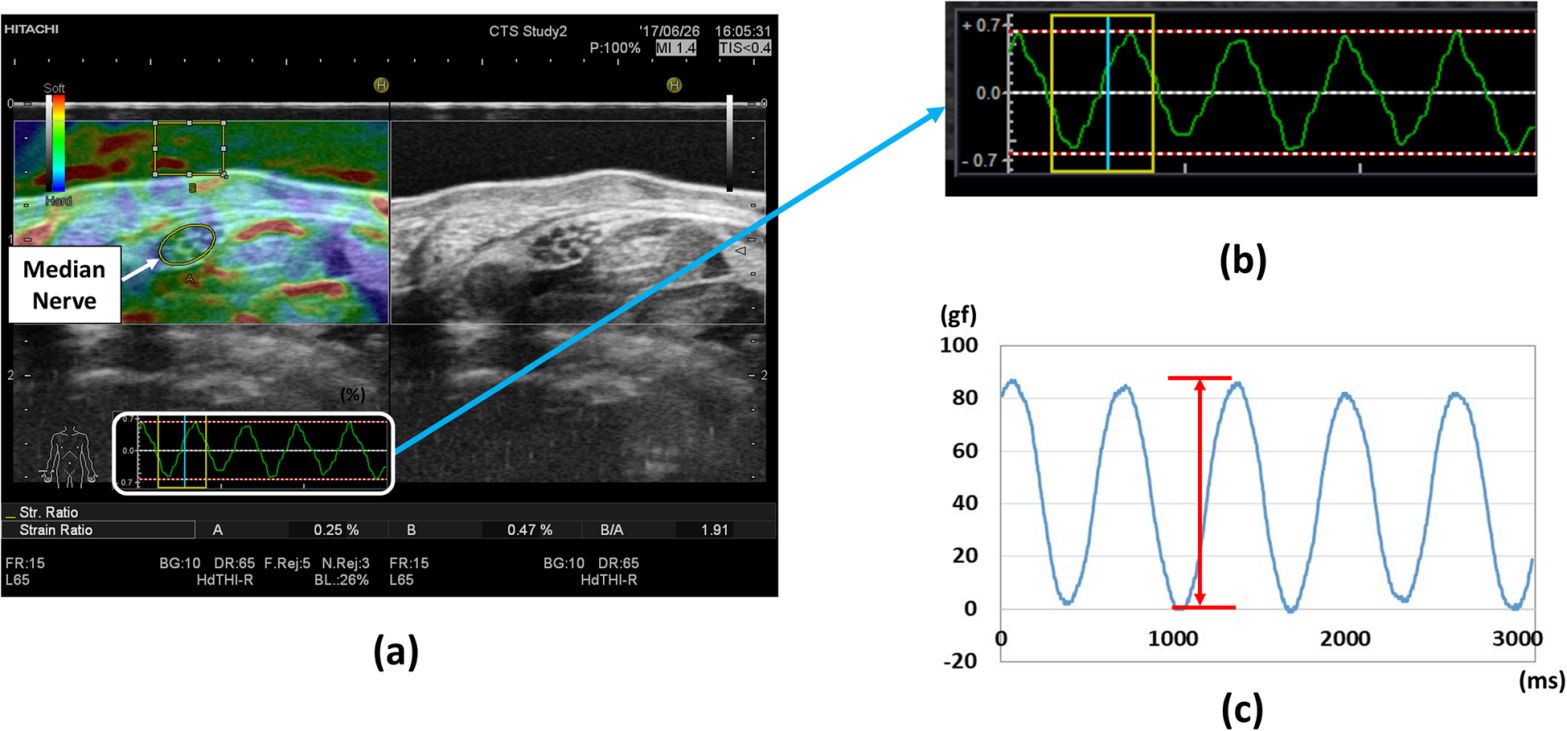
An example image of strain and pressure measurement. **a** Real-time Tissue Elastography mode of the ultrasound system. **b** An example of a strain waveform. **c** An example of a pressure waveform

The measurement was performed in the sitting position with the elbow mild flexion and the forearm supination position and the wrist neutral position (Fig. 1). The subject’s forearm was placed on the examination table. The transducer was placed parallel to the wrist crease (proximal carpal tunnel) and held perpendicular to the long axis of the forearm. The cross-sectional image of median nerve was displayed on the monitor. Median nerve strain was measured with the real time tissue elastography mode in the ultrasound system (Fig. 2-a). In order to avoid the effects of changes in the position of median nerve before and after carpal tunnel release, the measurements were performed with viewing the nerve in the center of the monitor, and the region of interest was set to fit within the nerve region. Vertical transducer movement of 1.0 mm was applied to the skin at a cycle of 1.5 Hz. Ultrasound images were taken with an acoustic coupler. The morphological changes of the median nerve in the direction of compression were evaluated as the strain (Fig. 2-b). The applied pressure to the skin was simultaneously recorded by the force processor. Based on the pressure when the transducer was placed on the skin, the force when the transducer pressed down the skin was evaluated as the applied pressure (Fig. 2-c). The median nerve strain (%) and applied pressure (gram force, gf) were measured five times, and the mean value of five times was used for further analysis. As a clinical elastic modulus, the pressure-strain ratio (gf /%) obtained with dividing the pressure by the strain was also evaluated.

### Measurement of median nerve morphology

In addition to the strain and applied pressure measurements, cross-sectional area of the median nerve was measured. The Image J Software (U. S. National Institutes of Health, Bethesda, USA) was used for the calculation of the cross-sectional area. B-mode image of the ultrasound was imported into the software. The median nerve was outlined and its area was calculated.

### Nerve conduction study

Neuropack MEB-2208 (Nihon Kohden Co., Tokyo, Japan) was used for the nerve conduction study. The nerve conduction studies were performed by an examiner who was not notified of clinical symptoms. An active electrode was placed over the motor point of the abductor pollicis brevis (APB) for the motor conduction study. The reference electrode was placed 3 cm distally to the active electrode. Median motor nerve stimulations were performed 7 cm proximal to the active electrode. The compound motor action potential on stimulation at the wrist was evaluated. For the sensory conduction study, the median nerve was examined antidromically. The active electrode was placed over the index finger, and the reference electrode was placed 3 cm distal to the active electrode. Median nerve stimulations were applied at 14 cm proximal to the active electrode. The sensory nerve action potential detected after the stimulation was evaluated. The nerve conduction studies were conducted while maintaining the room temperature at 24–26 degrees Celsius. The distal latencies of the motor and the sensory action potentials were used for further analysis. The distal latencies greater than 4.0 ms for the motor conduction study and 3.2 ms for the sensory conduction study were judged to be abnormal [24, 25].

### Statistical analysis

The ultrasound and electrophysiological parameters were evaluated before and three months after carpal tunnel release. Means and standard deviations were calculated using descriptive statistics. A paired t-test was used to compare the values before and after carpal tunnel release. *P* values of less than 0.05 were considered statistically significant. The correlations of the values between pressure/strain measurements and cross-sectional area of median nerve, distal motor latencies of nerve conduction studies were evaluated by the Pearson’s correlation coefficients with using pre- and post-operative measurements. Based on the patient recovery, the postoperative diagnostic potentials of the parameters were assessed by the receiver operating characteristic (ROC) curves. Before carpal tunnel release, the parameters were considered symptomatic examples. If a patient’s recovery was excellent or good after surgery, the parameters were considered non-symptomatic examples. If a patient’s recovery was fair or poor after surgery, the parameters remained symptomatic. Performances of the prognostic variables were quantified by calculating the area under the ROC curve (AUC). The cut-off values were defined by the points with the lowest distance to the upper-left corner of the ROC curves. All statistical analyses were performed using a standard software package (BellCurve for Excel version 2.12, SSRI Co., Tokyo, Japan).

## Results

**Fig. 3:**
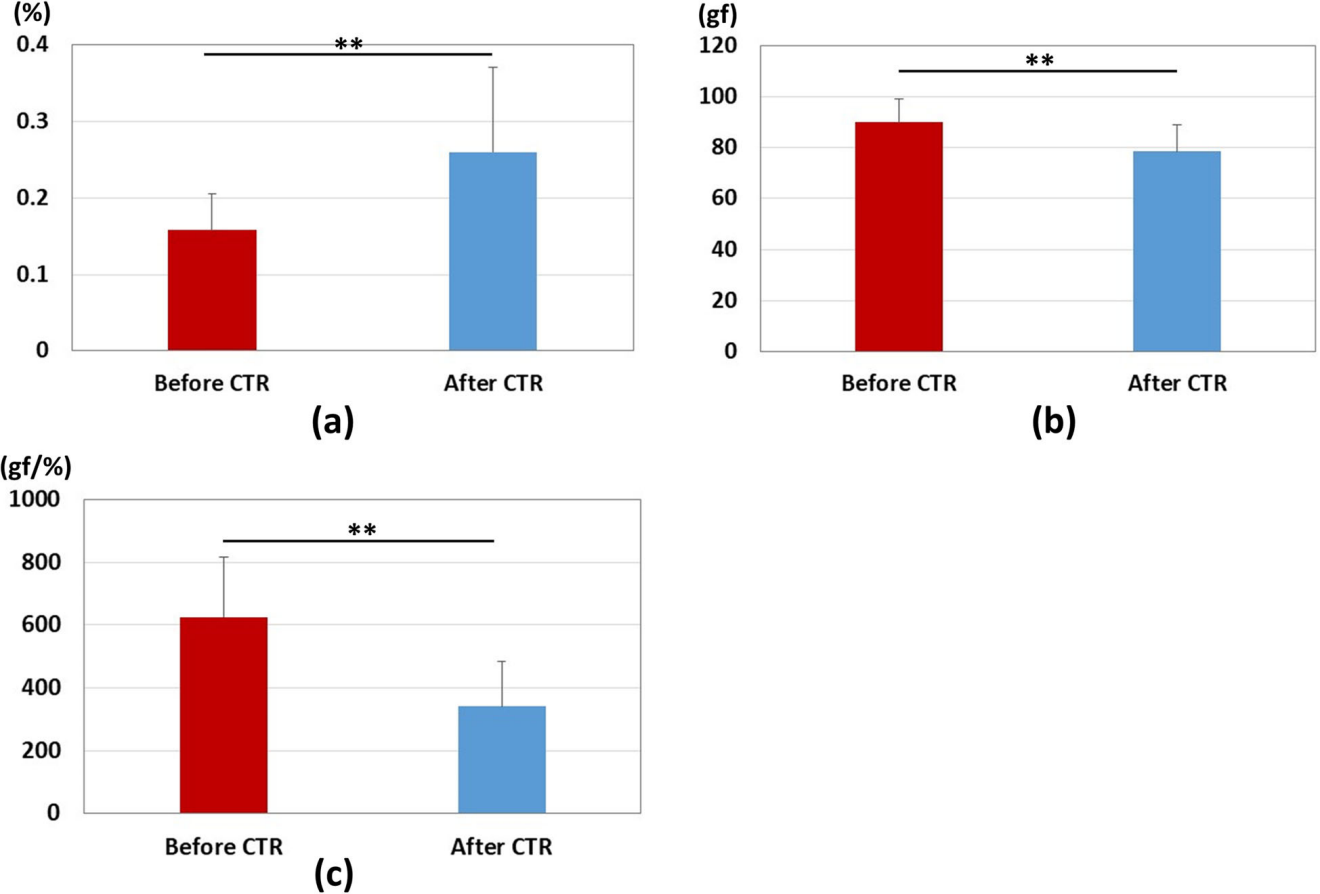
Results of strain, pressure, and pressure-strain ratio. Red bar: measurements before carpal tunnel release. Blue bar: measurements after carpal tunnel release. **: Significant difference before and after carpal tunnel release (*P* < 0.01). **a** Strain. **b** Pressure. **c** Pressure-strain ratio

**Fig. 4:**
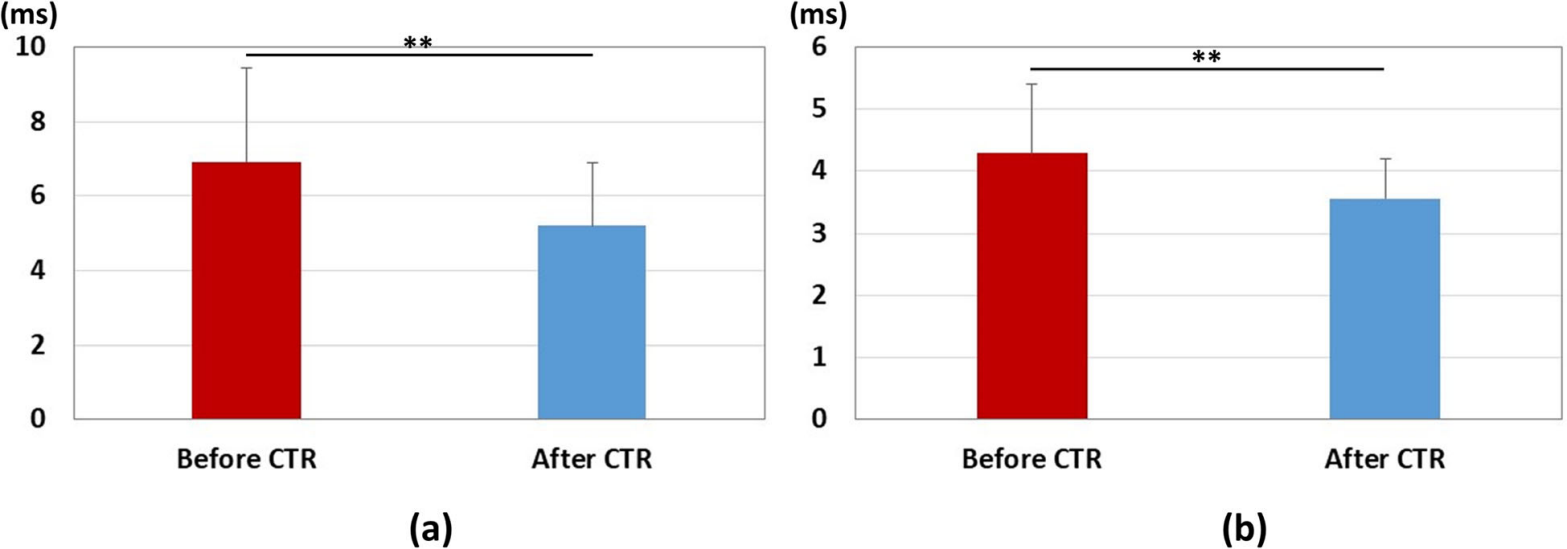
Results of nerve conduction study. Red bar: measurement before carpal tunnel release. Blue bar: measurements after carpal tunnel release. **: Significant difference before and after carpal tunnel release (*P* < 0.01). **a** Motor nerve conduction study, (**b**) Sensory nerve conduction study

**Fig. 5:**
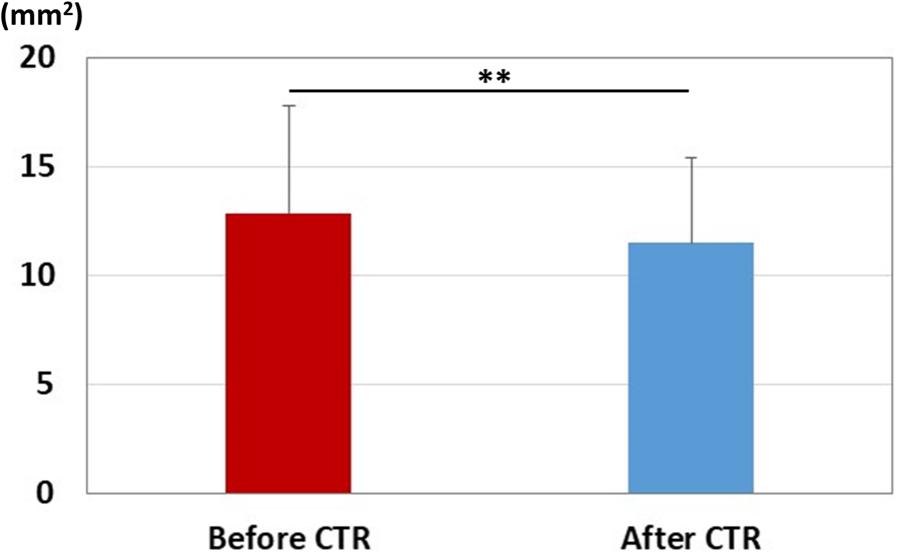
Results of cross-sectional area of median nerve. Red bar: measurement before carpal tunnel release. Blue bar: measurements after carpal tunnel release. **: Significant difference before and after carpal tunnel release (*P* < 0.01)

After carpal tunnel release, ten wrists showed excellent recovery, twenty-five wrists showed good recovery, nine wrists showed fair recovery, and one wrist showed poor recovery. Table 1 shows the summary results of each parameter. The results of median nerve strain, applied pressure, and pressure-strain ratio results are shown in Fig. 3. There was a significant increase in the strain after carpal tunnel release (*P* < 0.01), and significant decreases in the pressure and the pressure-strain ratio (*P* < 0.01). The results of distal latency for the motor nerve conduction study are shown in Fig. 4-a. Significant decreases in the distal latencies for both motor and sensory nerve conduction studies were observed after carpal tunnel release (*P* < 0.01). The results of cross-sectional area of median nerve are shown in Fig. 5. There was a significant decrease in the cross-sectional area of median nerve after carpal tunnel release (*P* < 0.01).

**Table 1:** Summary results of each parameter

	Before CTR			After CTR			
	Average (SD)	Median	Range	Average (SD)	Median	Range	
Strain (%)	0.16 (0.05)	0.16	0.07-0.32	0.26 (0.11)	0.24	0.10-0.55	**
Pressure (gf)	89.8 (9.5)	90.4	54.5-105.5	78.4 (10.5)	74.6	53.8-93.8	**
Pressure-strain ratio (gf/%)	625.4 (189.4)	568.0	249.3-1109.3	341.4 (143.9)	338.7	124.1-692.6	**
Motor latency (ms)	6.9 (2.5)	6.3	4.1-14.5	5.2 (1.7)	4.8	3.4-11.8	**
Sensory latency	4.3 (1.1)	4.2	2.9-6.9	3.6 (0.6)	3.5	2.7-4.9	**
Cross-sectional area (mm^2^)	12.9 (5.0)	12.0	6.0-24.9	11.5 (3.9)	11.4	5.4-20.6	**

**Significant difference before and after carpal tunnel release (*P* < 0.01)

**Fig. 6:**
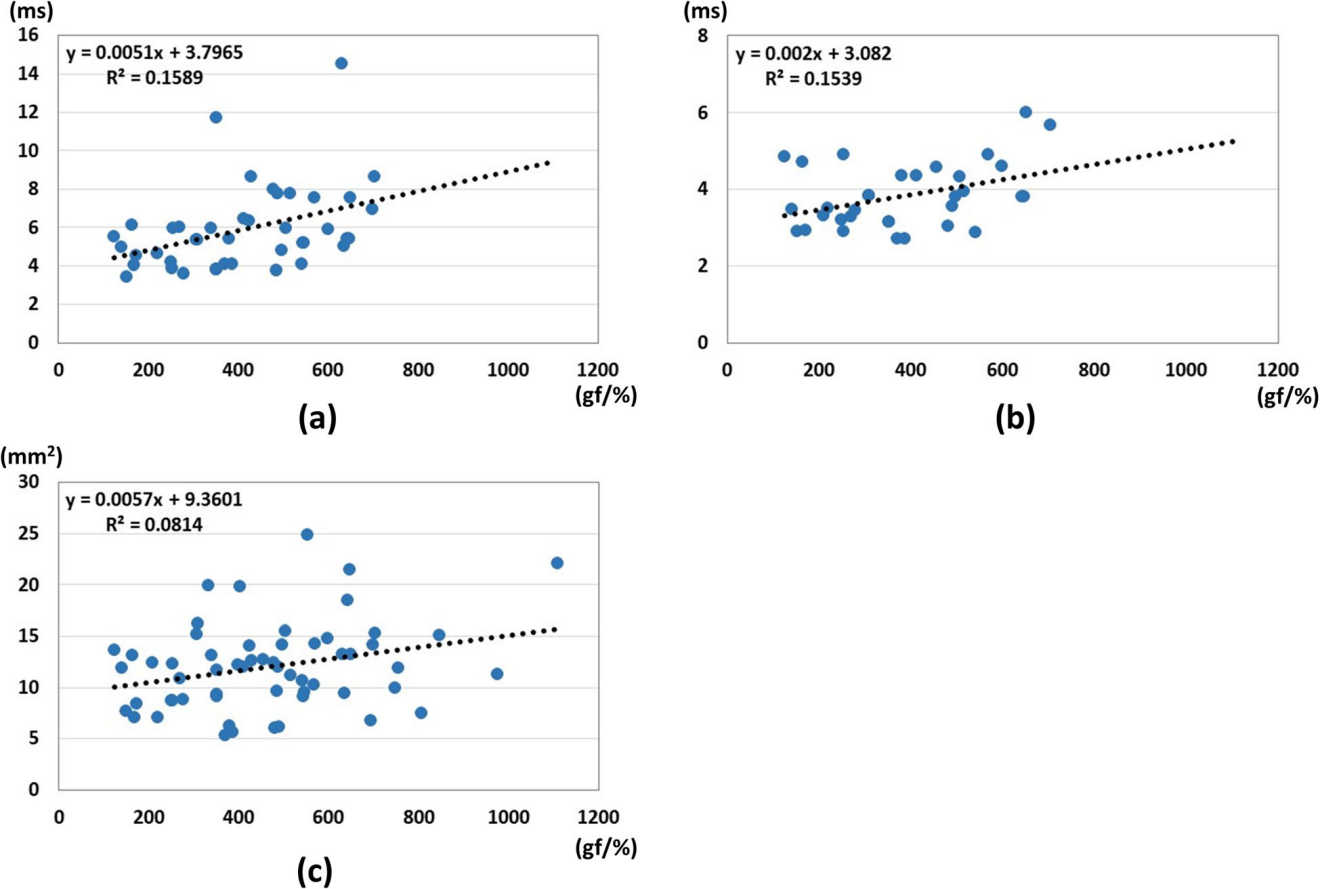
Results of correlations between pressure-strain ratio and other parameters. **a** Correlations between pressure-strain ratio and distal latency of motor nerve conduction study, (**b**) Correlations between pressure-strain ratio and distal latency of sensory nerve conduction study, (**c**) Correlations between pressure-strain ratio and median nerve area. There were no significant correlations among the parameters

The correlations between pressure-strain ratio and the nerve conduction studies, and between pressure-strain ratio and the cross-sectional area are shown in Fig. 6. In the results of correlation analysis, there were no significant correlations between the pressure-strain ratio and the other parameters. The correlation coefficients were 0.39, 0.39, and 0.29 for the correlation between the ratio and motor latency, the ratio and sensory latency, and the ratio and cross-sectional area, respectively.

**Fig. 7:**
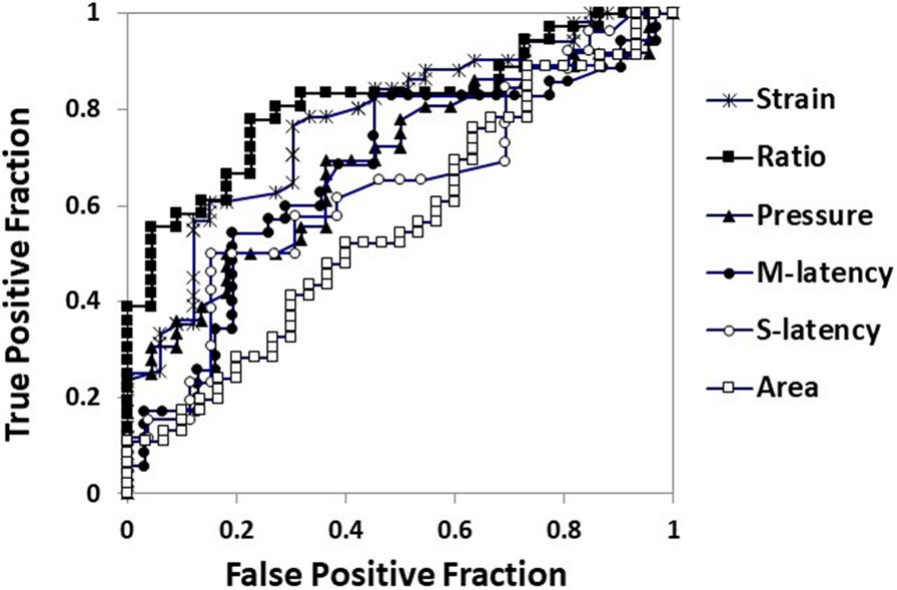
Results of receiver operating characteristic curve analysis. Triangle: pressure, asterisk: strain, black square: pressure-strain ratio, black circle: distal latency of motor nerve study, distal latency of sensory nerve conduction study, white square: cross sectional area of median nerve. The areas under the curves were 0.688, 0.788, 0.771, 0.650, 0.727, and 0.535 for the pressure, strain, pressure-strain ratio, motor latency, sensory latency, and area, respectively

The results of ROC curve analysis are shown in Fig. 7. The areas under the curves were 0.689, 0.773, 0.811, 0.668, 0.637, and 0.562 for the pressure, strain, pressure-strain ratio, motor latency, sensory latency, and cross-sectional area, respectively. Pressure-strain ratio had the highest AUC value, and the cross-sectional area had the lowest. The cut-off values for each parameter are shown in Table 2. The cut-off values for each parameter were 84.2 gf, 0.19, 410.9%/gf, 5.0 ms, 4.1 ms, and 11.9 mm^2^ for the pressure, strain, pressure-strain ratio, motor latency, sensory latency, and area, respectively.

**Table 2:** Results of cut-off values

	Cut-off values	False Positive Fraction	True Positive Fraction	Odds Ration
Strain (%)	0.19	0.303	0.765	7.48
Pressure (gf)	84.2	0.364	0.694	3.98
Pressure-strain ratio (gf/%)	410.9	0.227	0.778	11.90
Motor latency (ms)	5.0	0.452	0.829	5.87
Sensory latency	4.1	0.154	0.500	5.50
Cross-sectional area (mm^2^)	11.9	0.400	0.522	1.64

## Discussion

In the present study, we investigated the changes in the median nerve strain, application of pressure to the skin, distal latency of motor and sensory conduction studies, and cross-sectional area in CTS patients before and after carpal tunnel release. There was a significant increase in the strain and significant decreases in the applied pressure and the pressure-strain ratio, after carpal tunnel release. In addition, there were significant decreases in the distal latencies and the cross-sectional area. This suggests that carpal tunnel release affects not only functional and morphological recoveries, but also strain and surrounding tissue stiffness. This method and the result of strain/electrophysiological measurements may support understanding of median nerve recovery in relation to the stiffness of the surrounding structures.

Clinical significance of strain measurement has been widely accepted. However, questions remain regarding universality of the evaluation. This is because the ultrasonography is highly dependent on the examiner, and the load on the tissue during strain measurement has not been evaluated in previous studies [26]. These issues limit the reproducibility and universality of the strain measurement. In our previous studies, we tried to develop quantitative methods to measure strain of the tissues. It was found the ultrasonic strain measurements of the median nerve were superior to morphologic assessment in diagnosing CTS [27]. During the process, it was noticed applied pressure had also important factor to measure strain of the tissues. Then, the system was modified to measure strain and applied pressure simultaneously [14]. The method reported in this study may improve the reproducibility of the strain measurement as it measures strain and applied pressure simultaneously, with reproducible stress.

The results suggested that median nerve strain had a higher reliability to identify clinical recovery at three months after carpal tunnel release. Because electrophysiological recovery is associated with histological recovery, once the nerve denervates it may take longer to recover than tissue elasticity. Thus, strain measurement may be more reliable for clinical recovery than electrophysiological test assessment in the early stage after carpal tunnel release. From our previous study, median nerve strain for CTS patients with conservative treatment was 0.21% on average. ^14^ The strain of the patients for carpal tunnel release in this study was 0.16% on average. The strains were significantly lower in the patients with operative treatment compared with the patients with conservative treatment. This suggests that strain measurement may also be useful in deciding the indications for carpal tunnel release.

The measurement of the pressure reflects the internal pressure around the carpal tunnel. Carpal tunnel pressure in patients with CTS is reported to be higher than in healthy individuals. It was reported that elevated carpal tunnel pressure in CTS patients decreased to a normal level immediately after carpal tunnel release [28–30]. In this study, the applied pressure also decreased after carpal tunnel release. This may be due to changes in carpal tunnel compliance after carpal tunnel release. ^29^ Several reports measured carpal tunnel pressure clinically [15–21]. Constant infusion flow techniques were the most commonly used. Since this method requires the insertion of a slit catheter into the carpal tunnel, it was difficult to evaluate carpal tunnel pressure over time. The method introduced in this study may be a way to estimate the pressure around the carpal tunnel non-invasively.

The pressure-strain ratio showed the highest accuracy in terms of recognizing clinical recovery compared with other parameters. In our previous study, it was found that the pressure-strain ratio was the highest accuracy for the diagnosis of CTS [14]. The results of this study had the same tendency. Generally, the elasticity of a material is expressed using Young’s modulus, which is defined as the stress (force per unit area) divided by the strain (displacement caused by the force) of a material. The elastography strain is expressed by the subsequent tissue displacement tracked between pairs of echo frames, and the strain calculated from the axial gradient of the displacements. Since the strain is expressed by the rate of tissue displacement, the pressure-strain ratio cannot be an absolute indicator of elasticity. However, it still reflects the relationship between the strain and the applied pressure. This method could be used to evaluate tissue elasticity in the human body. As the pressure-strain ratio reflected the clinical recovery, it may be useful in determining the effectiveness of treatment.

There are several limitations to our study. Firstly, clinical recovery was defined by the qualitative assessment of the patient. Quantitative assessment of clinical recovery based on a questionnaire (i.e., DASH, Boston carpal tunnel syndrome questionnaire, etc.) may be required to compare parameters. In addition, the degree of clinical outcomes were heard by the surgeon. It may be likely that patients will be biased towards presenting more positive outcomes. Secondly, we measured the strain and pressure at the proximal carpal tunnel. Since the median nerve strain differs from proximal to distal, there may be some difference in the results at different levels of the carpal tunnel. In addition, we evaluated these parameter at only three months after the surgery. The results may change during the histological recovery of median nerve. Thirdly, the patient age distribution was heterogeneous. Aging may cause fibrosis and/or atrophy in tissues, so the age distribution may have affected the results. There was one patient relatively older who showed a poor clinical outcome. In the future study, we may need more attention to investigate the poor outcome patients. To evaluate prognostic values of strain and pressure measurements, it needs to measure at multiple time points and from larger groups of patients with various outcomes. Lastly, we did not compare the applied pressure to the internal pressure of the carpal tunnel. Because measurements were performed at outpatient clinic visits, the use of invasive measurements was avoided. To evaluate the relation between applied pressure and carpal tunnel pressure may require simultaneous pressure measurement. In addition, the complexity of the measurements should be simplify in the future study.

## Conclusions

In conclusions, this study investigated the postoperative diagnostic potentials of median nerve strain and applied pressure measurements to assess clinical recovery after carpal tunnel release. ROC curve analysis demonstrated that the median nerve strain and the pressure-strain ratio were highly associated with clinical recovery after carpal tunnel release. Pressure-monitor ultrasonography and the parameters used in this study may be useful to assess therapeutic effect during the process of carpal tunnel syndrome treatment.

## Data Availability

The datasets analyzed during the current study are available from the corresponding author on reasonable request.

## Abbreviations

AAEM: American association of electrodiagnostic medicine
APB: Abductor pollicis brevis
AUC: Area under the ROC curve
CTS: Carpal tunnel syndrome
DASH: Disabilities of the arm, shoulder and hand
ROC: Receiver operating characteristic
